# Closed–loop oxygen control improves oxygenation in pediatric patients under high–flow nasal oxygen—A randomized crossover study

**DOI:** 10.3389/fmed.2022.1046902

**Published:** 2022-11-16

**Authors:** Ozlem Sandal, Gokhan Ceylan, Sevgi Topal, Pinar Hepduman, Mustafa Colak, Dominik Novotni, Ekin Soydan, Utku Karaarslan, Gulhan Atakul, Marcus J. Schultz, Hasan Ağın

**Affiliations:** ^1^Department of Pediatric Intensive Care Unit, Dr. Behcet Uz Children’s Disease and Surgery Training and Research Hospital, Health Sciences University, İzmir, Turkey; ^2^Department of Medical Research, Hamilton Medical AG, Bonaduz, Switzerland; ^3^Department of Pediatric Intensive Care Unit, Erzurum Territorial Training and Research Hospital, Health Sciences University, Erzurum, Turkey; ^4^Department of Pediatric Intensive Care Unit, Cam Sakura Training and Research Hospital, Health Sciences University, Istanbul, Turkey; ^5^Department of Intensive Care, Amsterdam UMC, Academic Medical Center, Amsterdam, Netherlands; ^6^Tropical Medicine Research Unit, Mahidol University, Bangkok, Thailand; ^7^Nuffield Department of Medicine, University of Oxford, Oxford, United Kingdom

**Keywords:** intensive care, pediatric [MeSH], hypoxemia, oxygen therapy, high flow (NHF), automation, closed-loop, oxygen controller

## Abstract

**Background:**

We assessed the effect of a closed–loop oxygen control system in pediatric patients receiving high–flow nasal oxygen therapy (HFNO).

**Methods:**

A multicentre, single–blinded, randomized, and cross–over study. Patients aged between 1 month and 18 years of age receiving HFNO for acute hypoxemic respiratory failure (AHRF) were randomly assigned to start with a 2–h period of closed–loop oxygen control or a 2–h period of manual oxygen titrations, after which the patient switched to the alternative therapy. The endpoints were the percentage of time spent in predefined SpO_2_ ranges (primary), FiO_2_, SpO_2_/FiO_2_, and the number of manual adjustments.

**Findings:**

We included 23 patients, aged a median of 18 (3–26) months. Patients spent more time in a predefined optimal SpO_2_ range when the closed–loop oxygen controller was activated compared to manual oxygen titrations [91⋅3% (IQR 78⋅4–95⋅1%) vs. 63⋅0% (IQR 44⋅4–70⋅7%)], mean difference [28⋅2% (95%–CI 20⋅6–37⋅8%); *P* < 0.001]. Median FiO_2_ was lower [33⋅3% (IQR 26⋅6–44⋅6%) vs. 42⋅6% (IQR 33⋅6–49⋅9%); *P* = 0.07], but median SpO_2_/FiO_2_ was higher [289 (IQR 207–348) vs. 194 (IQR 98–317); *P* = 0.023] with closed–loop oxygen control. The median number of manual adjustments was lower with closed–loop oxygen control [0⋅0 (IQR 0⋅0–0⋅0) vs. 0⋅5 (IQR 0⋅0–1⋅0); *P* < 0.001].

**Conclusion:**

Closed-loop oxygen control improves oxygenation therapy in pediatric patients receiving HFNO for AHRF and potentially leads to more efficient oxygen use. It reduces the number of manual adjustments, which may translate into decreased workloads of healthcare providers.

**Clinical trial registration:**

[www.ClinicalTrials.gov], identifier [NCT 05032365].

## Introduction

Application of high–flow nasal oxygen (HFNO) has become a first–line therapy for acute hypoxemic respiratory failure (AHRF) in intensive care units (ICUs), emergency departments, and now also in pediatric patients (1–3). A recent worldwide survey demonstrated a large variability in HFNO settings, including the fraction of inspired oxygen (FiO_2_) (3).

Pediatric intensivists tend to avoid both hypoxemia and hyperoxemia (4–7) as previous studies suggest a relation between excessive or inadequate use of oxygen and mortality in pediatric ICU patients receiving oxygen therapy (8–12). While ideally partial pressure of arterial oxygen (PaO_2_) and arterial oxygen saturation (SaO_2_) are used to titrate oxygen, these values are often difficult to monitor in pediatric patients. Pulse oximetry (SpO_2_) may serve as an attractive alternative as it offers the advantage of continuous monitoring. A search in MEDLINE, Embase, CINAHL, and Web of Science on May 16, 2022, with the terms (“closed-loop” OR “automatic”) AND (“oxygen” OR “oxygen therapy”) with no date or language restrictions, identified 33 clinical investigations of which 27 were randomized clinical studies. All studies concluded that SpO_2_ can be used by closed-loop oxygen systems to automatically adjust the FiO_2_. None of these studies, however, assessed the effects of closed-loop oxygen control during HFNO in pediatric patients (13–44).

Studies testing the efficiency and safety of closed-loop oxygen systems in pediatric patients receiving HFNO for AHRF are currently lacking. Therefore, we performed a randomized crossover study to evaluate the efficiency of a closed-loop oxygen control system integrated into a HFNO device with respect to the quality of oxygen therapy in pediatric patients. We also tested its safety, determined total oxygen use, and compared the number of manual adjustments between closed-loop oxygen control and manual oxygen titration. We hypothesized this closed-loop oxygen system to increase time spent within predefined optimal SpO_2_ ranges.

## Materials and methods

### Study design

This is a multicentre, single-blinded, randomized, crossover study of closed-loop oxygen control vs. manual oxygen titrations in pediatric patients in three hospitals in Turkey. Patients were screened for participation in the pediatric ICUs in the Dr. Behcet Uz Children’s Research and Training Hospital in Izmir, the Erzurum Territorial Training and Research Hospital in Erzurum, and the Cam Sakura Research and Training Hospital in Istanbul, from September 2021 to January 2022. The study was approved by the Institutional Review Boards (604/2021/13-01) and conducted in accordance with the Declaration of Helsinki. The study is registered at ClinicalTrials.gov (study identifier NCT 05032365).

### Participants

Patients were eligible if: (1) aged between 1 month and 18 years of age and (2) receiving HFNO with FiO_2_ ≥ 25% to maintain SpO_2_ within the preferred target ranges. Patients were only included if considered to be in a clinical stable condition, i.e., not expected to need a change in respiratory support, like non-invasive or invasive ventilation in the next 5 h. We excluded patients with congenital or acquired hemoglobinopathies affecting SpO_2_, patients with cyanotic heart disease, and patients who needed continuous infusion of epinephrine or norepinephrine at rates higher than 0.2 μg/kg per minute. We also excluded patients for which no written informed consent could be obtained, patients that were previously included in this study, and patients that were enrolled in another interventional study.

### Randomization and masking

Patients were randomized to start with a 2–h period of closed-loop oxygen control or a 2–h period of manual oxygen titration. Thereafter, patients were switched to the alternate therapy. Randomization was 1:1, with blocks of four, using sealed opaque envelopes. Due to the intervention, healthcare staff could not be blinded. Patients remained blinded for the way oxygen was titrated.

### Procedures

Patients were equipped with an appropriately sized and placed nasal cannula and kept in a semi-recumbent position for the duration of the study. A pediatric ventilator equipped with a humidifier device (Hamilton–C1 with H–900 humidifier, Hamilton Medical AG, Bonaduz, Switzerland) was used for HFNO. Patients were sedated as needed, at a sedation level sufficient for each patient. The sedation level was not altered for the duration of the study. Patient care and standard activities, such as suctioning of secretions or feeding, were uninterruptedly performed as usual and at random in either period. At the study sites, during daytime and night-time shifts, the nurse and doctor to patient ratios were approximately 6:1 and 12:1, and 2:1 and 3:1, respectively. This setting not changed during the conduct of the study, i.e., these ratios were not different during the two crossover phases. Also, there was no study personnel present during these two phases.

After randomization, the attending physician decided on the optimal SpO_2_ range, individualized for each patient according to the current clinical situation and medical history. After the first 2–h period, a washout period was established for 30 min, after which the patient was switched to the second 2–h period with the alternate oxygen titration strategy (Supplementary Figure 1). With closed-loop oxygen control, patients’ SpO_2_ was kept in a predefined target range via automatic adjustment of the FiO_2_. In the manual oxygen titration phase of the study, manual adjustments to FiO_2_ were performed by the bedside doctors or nurses, using the same SpO_2_ target range. The flow of the HFNC was not altered during the two crossover phases of the trial. The SpO_2_ target range was defined by setting four cutoffs: an upper and a lower “optimal” cutoff, and an upper and lower “suboptimal” cutoff. The optimal cutoffs were from 94 to 98% or from 92 to 96%. The corresponding suboptimal cutoffs were from 90 to 94% and from 98 to 99%, and from 88 to 92% and 96 to 98% (Supplementary Table 1). The running principles of the closed-loop system are detailed in Supplementary Table 2.

### Data collection

Ventilation parameters were captured in a case report form (CRF). HFNO data, including FiO_2_, airflows, and manual titrations were captured every second using a Memory Box (Hamilton Medical AG) connected to the RS–232 interface port on the ventilator.

### Definitions

Every recorded value of SpO_2_ was classified as either optimal if within the individualized predefined range, suboptimal high or low when outside the optimal SpO2 range, but inside the suboptimal cut-offs, or unacceptable when beyond the suboptimal cut-offs (as shown in Supplementary Table 1).

### Outcomes

The primary objective of the study was to assess the efficiency of the closed-loop oxygen control system. Therefore, the primary endpoint was the percentage of time spent in predefined target ranges for SpO_2_ in each 2–h period. Secondary endpoints were the percentage of time spent in suboptimal and unacceptable SpO_2_ ranges, the FiO_2_ and PaO_2_/FiO_2_ ratio, the number of manual oxygen adjustments, and the number of alarms.

### Power calculation

The sample size was calculated by means of a pilot study of seven patients (7 × 240 = 1,680 min) in which we determined the difference in the percentage of time spent in optimal target ranges for SpO_2_ between closed-loop and manual oxygen titrations. Based on the pilot data, G*Power computed that the study should have an additional 21 patients to detect an effect size of Cohen’s *d* = 0.86 with 95% power (two-tailed α of 0.05) in a Wilcoxon signed-rank test (45). To account for potential dropouts, defined as a patient who required either non-invasive ventilation or intubation for invasive ventilation during the two phases of the study, consent withdrawal by patient or family, poor quality of SpO_2_ readings for > 1 h during one of the study phases, or technical problem in recording, we decided to have a sample size of 23 patients.

### Statistical analysis

Shapiro–Wilk, skewness, and kurtosis normality tests were used to check the distribution of data. Continuous data were expressed in terms of either mean and standard deviation (SD) or median and interquartile range [IQR], according to the distribution.

Data were analyzed using either a paired samples *t*-test or Wilcoxon test, depending on which was most appropriate. The Wilcoxon signed-rank test was used for the comparison between the percentage of time spent in the target range of SpO_2_ with manual FiO_2_ adjustments and the percentage with closed-loop FiO_2_ control.

A *P*-value of less than 0⋅05 was considered statistically significant for all comparisons. Data were calculated with MATLAB (version 2021b) (The MathWorks, Inc., Natick, MA, United States) and statistical testing was carried out with the XLSTAT (version 2016) (Addinsoft, Paris, France). Figures were constructed using JASP (version 2022) (JASP Team, Amsterdam, The Netherlands) and GraphPad PRISM (version 9) (San Diego, CA, USA).

## Results

**FIGURE 1:**
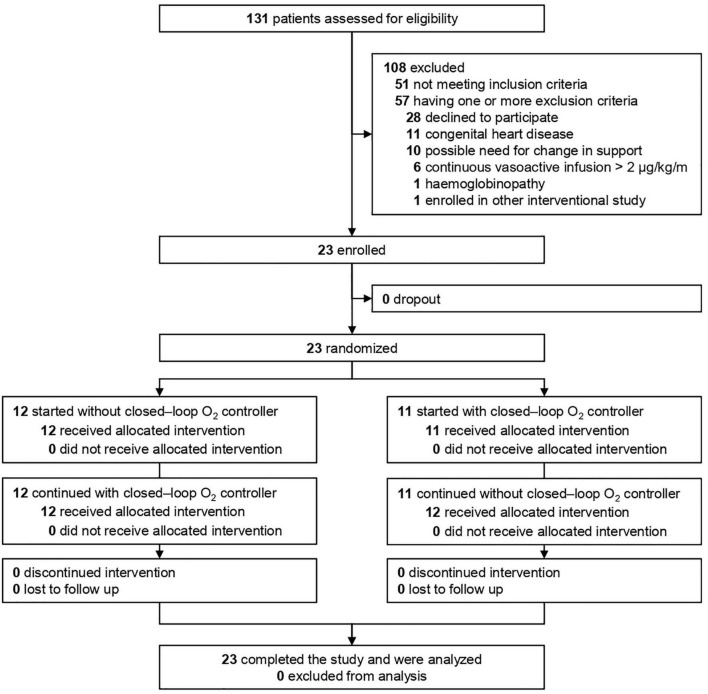
Trial profile.

From August 2021 to November 2021, 131 patients were screened; of 80 eligible patients, 57 met one or more exclusion criteria, and 23 patients were included (Figure 1). Baseline characteristics are presented in Table 1. The majority of patients was aged 2 years of age or younger, and in approximately half of the patients, AHRF was due to a respiratory infection. Two-third of patients received HFNO after having received invasive ventilation.

**TABLE 1:** Baseline characteristics of the study cohort.

Variables	Median (IQR) or mean (*SD*) or *n* (%)
Gender ratio (%f/%m)	43/57
Age (months)	18 (3–26)
Height (cm)	75 (55–87)
IBW (kg)	9.3 (4.5–12.2)
PIM3	10 (6.3–18.2)
PELOD	5.5 (3.5–19.2)
PICU duration (days)	4 (3–18)
S/F ratio	242.6 (207.6–312)
**HFNO settings**	
Flow (l/m)	2 (1.6–2.4)
Temperature (°C)	37 (37–38)
**Reason for HFNO**	
After extubation	15 (65)
*De novo* ventilation support	8 (35)
**Admission diagnosis**	
**Respiratory**	
*A. pneumonia*	11 (48)
*A. bronchiolitis*	
*Cystic fibrosis*	
*LRTI*	
*Laryngotracheomalacia*	
**Neurologic**	4 (17)
*SE*	
*Meningoencephalitis*	
*Hyrdrocephalus*	
**Cardiovascular**	
PDA	3 (13)
AVSD	
VSD	
**Sepsis**	3 (13)
**Renal/metabolic**	
RTA	2 (9)
DKA	
**Lung physiology**	
Obstructive	13 (57)
Restrictive	4 (17)
Mixed	6 (26)

Data are expressed as median (interquartile range, IQR) or as mean (standard deviation, SD) or number and percentage. IBW, Ideal body weight; PIM3, Pediatric index of mortality 3, probability of death; PELOD, Pediatric logistic organ dysfunction, probability of death; PICU duration, duration of PICU stay until study day; S/F ratio, Peripheral oxygen saturation/fraction of inspired oxygen; *A. pneumonia*, Acute *pneumonia*; *A. bronchiolitis*, Acute bronchiolitis; LRTI, Lower respiratory tract infection; SE, Status epilepticus; VP Shunt, Ventriculoperitoneal shunt; VSD, Ventricular septal defect; PDA, Patent ductus arteriosus; AVSD, Atrioventricular septal defect; RTA, Renal tubular acidosis; DKA, Diabetic Keto Acidosis.

**FIGURE 2:**
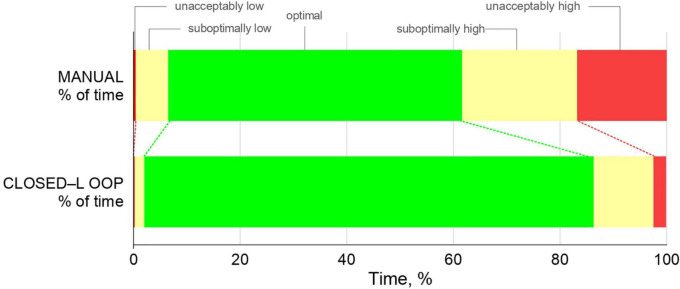
Time spent in optimal, suboptimal, and unacceptable SpO_2_ ranges.

Patients spent more time in optimal SpO_2_ ranges when the oxygen controller was activated compared to manual oxygen titrations [91⋅3% (IQR 78⋅4–95⋅1%) vs. 63⋅0% (IQR 44.4–70.7%), mean difference 28⋅2% (95%–CI 20⋅6–37⋅8)]; *P* < 0.001] (Table 2 and Figure 2).

**TABLE 2:** Primary and secondary outcomes.

Variable	Closed loop	Manual	Median difference (95% CI)	*P*-value
***Primary outcome***				
**Time spent in optimal SpO_2_ range** (%)	91.3 (78.4–95.1)	63 (44.4–70.7)	28.2 (20.6–37.8)	<0.001
***Secondary outcomes***				
**Time spent in suboptimal SpO_2_ range** (%)				
Low	1.5 (0.6–2.8)	2.6 (0.2–8.8)	–2.9 (–6 to 0.2)	0.086
High	5.9 (3.4–15.1)	19.9 (7.7–37.5)	–9.7 (–16.8 to –4.1)	0⋅003
Mean FiO_2_ (%)	33.3 (26.6–44.6)	42.6 (33.6–49.9)	–4.3 (–8⋅7 to 0.5)	0.07
Mean SpO_2_/FiO_2_	289.4 (206.7–348.3)	229.3 (195.9–295.3)	37.5 (6.5–70.5)	0.023
Manual adjustments (n/h)	0 (0–0)	0.5 (0–1)	–1.3 (–2.6 to –0.5)	<0.001
Alarms (n/h)	0 (0–0.3)	0.5 (0–2)	–1.2 (–2.3 to –0.4)	0.002
Percentage of time SpO_2_ available	98.5 (95.8–100)	97.2 (85.1–99.6)	2.6 (–0.2 to 7.7)	0.065
Percentage of time SpO_2_ < 88%	0 (0–0)	0 (0–0)	0 (0–0)	0.286
Percentage of time SpO_2_ < 85%	0 (0–0)	0 (0–0)	0 (0–0)	0.218
Number of events SpO_2_ < 88%	0 (0–0.5)	0.4 (0–1)	–0.4 (–0.9 to 0.1)	0.065
Percentage of time FiO_2_ < 40%	81.1 (42.6–1)	34.4 (0–1)	32.4 (–5⋅2 to 62)	0.103
Percentage of time 40% ≤ FiO_2_ ≤ 60%	10.3 (0–3.7)	29.6 (0–1)	–36 (–68⋅1 to –8.3)	0.015
Percentage of time FiO_2_ > 60%	0 (0–15.1)	0 (0–3.3)	13 (–8.7–53.1)	0.262

Data are expressed as median (interquartile range, IQR) or as mean (standard deviation, SD). Wilcoxon or student’s *t*-test was performed depending on each variable distribution according to the Shapiro–Wilk test. 95% CI, 95% confidence interval; SpO_2_, peripheral oxygen saturation; FiO_2_, Fraction of inspired oxygen.

Patients spent significantly less time in the unacceptably high and unacceptably low SpO_2_ ranges, and significantly less time in suboptimal low and suboptimal high SpO_2_ ranges when the oxygen controller was activated (Table 2 and Figure 2).

The median FiO_2_ was lower with closed-loop oxygen control, although this difference did not reach statistical significance (Table 2). The median SpO_2_/FiO_2_ ratio was significantly higher with closed-loop oxygen control (Table 2 and Supplementary Figure 2). There were significantly fewer manual adjustments needed, and the number of alarms was lower with closed-loop oxygen control (Table 2 and Supplementary Figure 2).

## Discussion

The findings of this multicentre randomized crossover study in pediatric patients under HFNO for AHRF can be summarized as follows: (1) Compared to manual oxygen titrations, the use of a closed-loop oxygen controller built into an HFNO device increased time spent in optimal SpO_2_ ranges; (2) decreased time spent in suboptimal and unacceptably high and unacceptably low ranges; (3) decreased time spent in suboptimal and unacceptable SpO_2_ ranges; (4) reduced oxygen use; and (5) lead to fewer manual adjustments and less alarms.

Our study has several strengths. We used a crossover design, making it possible to compare oxygen therapy under closed-loop oxygen control vs. manual oxygen titrations within each individual patient, increasing the statistical power of our investigation. We performed the study in multiple centers, including university hospitals and teaching hospitals, whereby we increased the external validity of the findings. We followed a strict study protocol and used randomization to prevent potential bias. We had an analysis plan in place before cleaning and closing the database; specifically, we predefined the optimal, suboptimal, and unacceptable SpO_2_ ranges that were based on consensus before the study. To our best knowledge this is the first study that tested the efficiency of closed-loop oxygen control in pediatric patients receiving HFNO.

The findings of our study are in line with previous studies testing the efficacy of closed-loop oxygen control in preterm infants (13–34) and adults (16, 35–44) receiving HFNO for hypoxemia of various causes. In all those studies, closed-loop oxygen control outperformed healthcare workers performing manual oxygen titrations with respect to the percentage of time spent in target SpO_2_ ranges and to the time spent in potentially dangerous SpO_2_ zones (13–44). Our study increases the understanding of the efficacy of closed-loop oxygen control in children with AHRF by providing proof that it also outperforms manual FiO_2_ titration in patients that are difficult to stabilize. In this respect, the performance is particularly advantageous since it has a strong potential to reduce workloads by the often overtasked ICU healthcare professionals (46, 47).

The findings of our investigation are also in line with studies of closed-loop oxygen controllers in invasively ventilated neonates (17, 48–50) and adult patients (51–56). In those studies, closed-loop oxygen control outperformed manual oxygen titrations with respiratory support in which oxygenation is not only driven by FiO_2_, but also by the provided tidal volumes and airway pressures. Taken together, closed-loop oxygen control has a wide range of applications in critically ill hypoxemic patients, under various forms of support, from non-intubated patients to intubated patients, from passive to active patients, and in patients that develop hypoxemia after extubation.

It is well known that intensive care doctors and nurses avoid both hypoxemia and hyperoxemia for various valid reasons. This is particularly true for healthcare workers treating critically ill neonates and children (4–7). This approach requires not only well-trained nurses but also large numbers of ICU nurses at the bedside. Indeed, hypoxemia can only be minimized if a nurse is near constantly present to perform manual oxygen titrations (48, 49). This can be very impractical and costly, and can actually not be delivered on a regular basis.

One previous study found that doctors’ responses to oxygen levels beyond the optimal SpO_2_ zones are “asymmetrical”; they try to prevent both hypoxemia and hyperoxemia, but they seem to put more emphasis on preventing the first than the second (57). Not surprisingly, this results in more time spent in suboptimal or unacceptable higher SpO_2_ ranges. The advantage of a closed-loop oxygen control system is that deteriorations to both lower and higher SpO_2_ ranges are prevented equally.

We found that SpO_2_/FiO_2_ ratios were higher under closed-loop oxygen control, suggesting that closed-loop oxygen control not only prevents hypoxemic and hyperoxemic deteriorations, but improves oxygenation overall. At the same time, it consumes less oxygen to achieve the same oxygenation levels, which may be critical in low-resource environments or at times of increased demand, such as a rise in oxygen use during a pandemic. This is consistent with several other research demonstrating that patients received less FiO_2_ under closed-loop control of FiO_2_ than during manual titration of FiO_2_ (49, 51, 52, 58).

Interestingly, we noticed much fewer manual adjustments and also much less alarms per hour with closed-loop oxygen control. This may also translate into a reduction in workloads as our data show that manual adjustments are hardly needed with the use of an oxygen controller. Higher workloads for ICU staff are associated with higher mortality rates (59). On top of that, our data also show a strong reduction in the number of alarms, which may increase patient comfort and sleep hygiene, and thereby reduce the risk of delirium (60, 61).

Our study has limitations. Due to the nature of the intervention, we were not able to blind the healthcare workers. However, we used predefined SpO_2_ zones that reflected the zones to which ICU nurses titrate FiO_2_ in daily practice. The periods of manual oxygen titrations and automated oxygen control were lasting only 2 h, and thus did not cover all daily activities. However, we wanted to have comparable patient conditions in the two crossover phases for as much as possible, and these conditions could change rapidly in pediatric patients. Additionally, we intended the two study phases to take place in a single shift, which limited the amount of time we had for the full investigation in each individual patient. The crossover design prohibits us from examining the effects of closed-loop oxygen control on clinical endpoints like duration of HFNO or escalation of ventilatory support to invasive ventilation. Future studies should focus on these endpoints.

## Conclusion

In conclusion, compared to manual oxygen titrations, closed-loop oxygen control improves time spent in favorable SpO_2_ zones and reduces time spent in potentially dangerous SpO_2_ zones in pediatric patients under HFNO for AHRF. In addition, closed-loop oxygen control improves overall oxygenation, uses less oxygen, and is associated with fewer manual adjustments and less alarms.

## Data availability statement

The raw data supporting the conclusions of this article will be made available by the authors, without undue reservation.

## Ethics statement

The studies involving human participants were reviewed and approved by the Ethical Committee of Dr. Behcet Uz Children’s Research and Training Hospital. Written informed consent to participate in this study was provided by the participants’ legal guardian/next of kin.

## Author contributions

OS, GC, ST, PH, MC, ES, UK, DN, and HA conceived and designed the study. OS, GC, ST, PH, MC, MS, ES, UK, and GA acquired and analyzed the data. GC, MS, GA, and HA interpreted the data. GC and HA did the statistical analysis. OS, GC, DN, MS, and HA drafted the manuscript. OS, GC, ST, PH, MC, ES, UK, and HA had full access to all of the data. All authors critically revised the manuscript for important intellectual content, were responsible for the final decision to submit for publication, and approved the manuscript.

## Supplementary material

Supplementary Figure 1 Trial flow diagram.

Supplementary Figure 2 Effect of closed-loop oxygen control on alarms, manual titrations, and SpO2/FiO2.
