# Supplementary figures and images for: Closed–loop oxygen control improves oxygenation in pediatric patients under high–flow nasal oxygen—A randomized crossover study
Source: Front Med (Lausanne). 2022 Nov 16;9:1046902. doi: 10.3389/fmed.2022.1046902 (PMC9708705; doi:10.3389/fmed.2022.1046902)

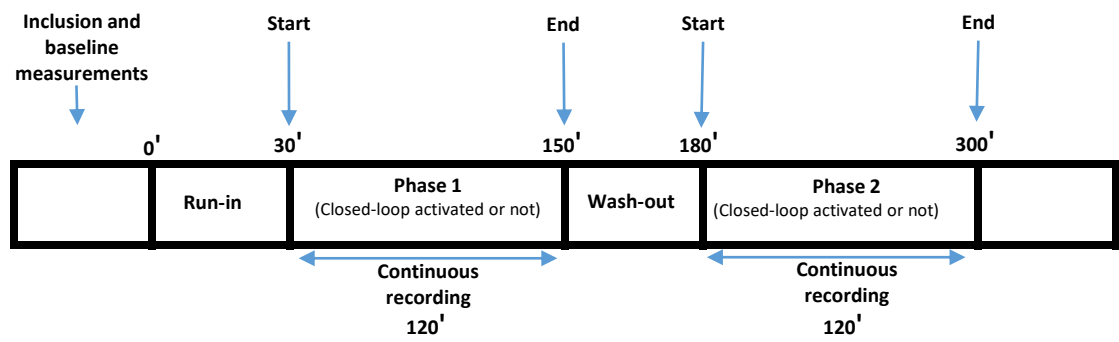

Supplement: Supplementary Figure 1 — Trial flow diagram. [file Data_Sheet_1.PDF]

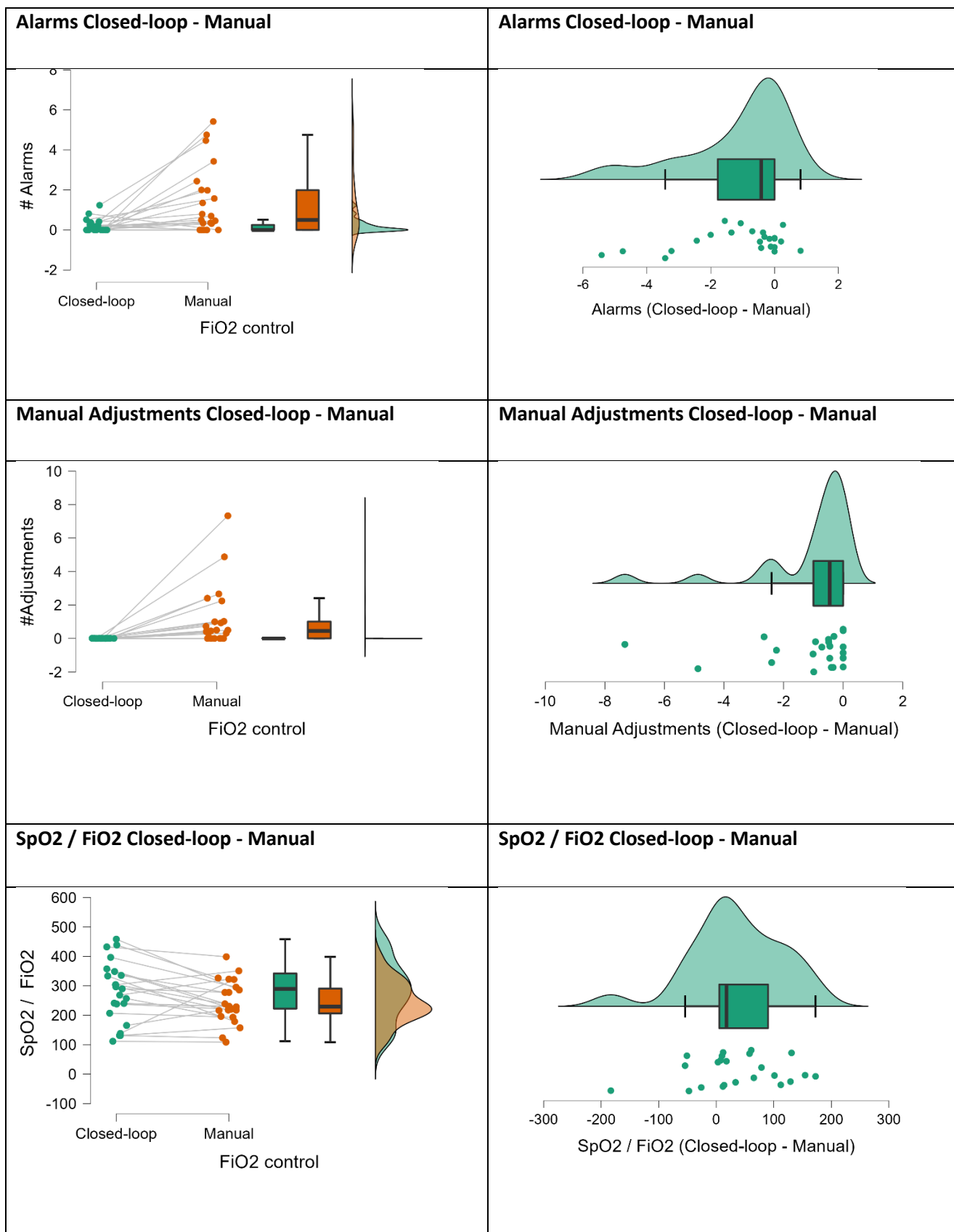

Supplement: Supplementary Figure 2 — Effect of closed-loop oxygen control on alarms, manual titrations, and SpO2/FiO2. [file Data_Sheet_2.PDF]
