# Supplementary material for: Closed–loop oxygen control improves oxygenation in pediatric patients under high–flow nasal oxygen—A randomized crossover study
Source: Front Med (Lausanne). 2022 Nov 16;9:1046902. doi: 10.3389/fmed.2022.1046902 (PMC9708705; doi:10.3389/fmed.2022.1046902)
Supplement: Supplementary file 3 [file Data_Sheet_3.PDF]

Supplementary Table 1, SpO2 (Peripheral oxygen saturation) predefined target ranges

Group	Unacceptably low	Suboptimally low	Optimal	Suboptimally high	Unacceptably high
**Higher Clinical Target**	< 90%	≥ 90% and < 94%	≥ 94% and ≤ 98%; > 98% if FiO2 = 0·21	> 98% and ≤ 99%	100%
**Lower Clinical Target**	< 88%	≥ 88% and < 92%	≥ 92% and ≤ 96%; > 96% if FiO2 = 0·21	> 96% and ≤ 98%	> 98

FiO2: Fraction of inspired oxygen
