# Supplementary material for: Closed–loop oxygen control improves oxygenation in pediatric patients under high–flow nasal oxygen—A randomized crossover study
Source: Front Med (Lausanne). 2022 Nov 16;9:1046902. doi: 10.3389/fmed.2022.1046902 (PMC9708705; doi:10.3389/fmed.2022.1046902)
Supplement: Supplementary file 4 [file Data_Sheet_4.PDF]

Supplement Table 2. Running principles of the closed–loop system.

IF	THEN
SpO_2 is in range (between Target-High and Target-Low)	The controller fine-tunes the Oxygen setting to get the patient's SpO_2 to the middle of the target range.
SpO_2 is low (below Target-Low and above Emergency-Low)	The controller increases the Oxygen setting every 30s.
SpO_2 is too low (below Emergency-Low)	The controller increases the Oxygen setting with larger step every 30s.
SpO_2 is high (above Target-High and below Emergency-High)	The controller decreases the Oxygen setting every minute.
SpO_2 is too high (above Emergency-High)	The controller decreases the Oxygen setting with larger step every minute.
SpO_2 measurement is unavailable	The Oxygen control is frozen and is displayed as a red circle together with an alarm
Closed-loop oxygenation controller is a rule based, proportional integral controller calculates the difference between the actual SpO_2 measurement and the target SpO_2 range set by the clinician in order to determine the treatment action. Automatic FiO2 relies on the SpO_2 measurements provided by a pulse-oximetry using a finger or ear probe. Abbreviations: SpO2: peripheral oxygen saturation; FiO2: fraction of inspired oxygen)	
